# Automated Detection of Red Lesions Using Superpixel Multichannel Multifeature

**DOI:** 10.1155/2017/9854825

**Published:** 2017-04-23

**Authors:** Wei Zhou, Chengdong Wu, Dali Chen, Zhenzhu Wang, Yugen Yi, Wenyou Du

**Affiliations:** ^1^College of Information Science and Engineering, Northeastern University, Shenyang, Liaoning 110004, China; ^2^Faculty of Robot Science and Engineering, Northeastern University, Shenyang, Liaoning 110004, China; ^3^School of Software, Jiangxi Normal University, Nanchang, Jiangxi 330022, China

## Abstract

Red lesions can be regarded as one of the earliest lesions in diabetic retinopathy (DR) and automatic detection of red lesions plays a critical role in diabetic retinopathy diagnosis. In this paper, a novel superpixel Multichannel Multifeature (MCMF) classification approach is proposed for red lesion detection. In this paper, firstly, a new candidate extraction method based on superpixel is proposed. Then, these candidates are characterized by multichannel features, as well as the contextual feature. Next, FDA classifier is introduced to classify the red lesions among the candidates. Finally, a postprocessing technique based on multiscale blood vessels detection is modified for removing nonlesions appearing as red. Experiments on publicly available DiaretDB1 database are conducted to verify the effectiveness of our proposed method.

## 1. Introduction

Diabetic retinopathy (DR) is one of the most serious performances of diabetic and the majority of people suffering from diabetes mellitus for more than ten years will eventually develop DR [1]. Moreover, with the development of the disease, it will cause vision loss [2]. Regular follow-up has been shown to help patients delay the progression of blindness and visual loss. Digital color fundus photography owns its low-cost and patient friendliness which is a prerequisite for large scale screening [3]. However, in DR screening program, the limited number of specialists cannot keep up with the rapid increasing number of DR patients. Under this circumstance, developing an automatic detection technique based on fundus images becomes extremely essential and urgent [4].

**Figure 1:**
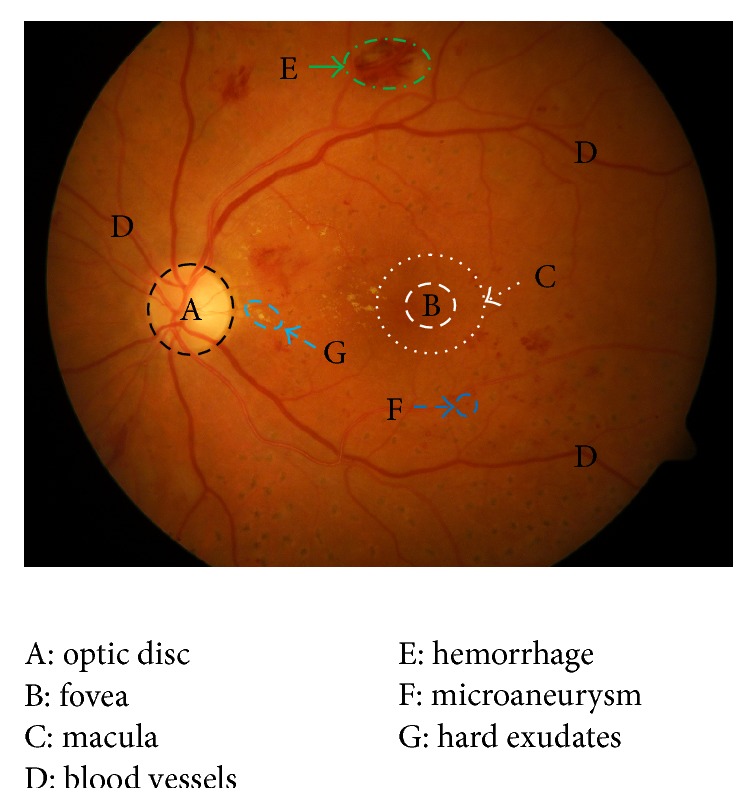
A retinal image with different types of lesions and main anatomical features.

There are several different components in retinal images (see Figure 1), such as blood vessels, fovea, macula, and optic disc. In clinical, ophthalmologists classify DR into two primarily phases, namely, nonproliferative DR (NPDR) and proliferative DR (PDR) [5]. NPDR can be regarded as the initial phase of DR. During this phase, the blood vessels become thin and leak fluid onto the retina [5]. Several lesions such as red lesions (microaneurysms and hemorrhages; see Figure 1), yellowish or bright spots (hard and soft exudates; see Figure 1), and interretinal microvascular abnormalities (IRMA) [6] can be produced during the phase of NPDR. The second phase of DR is the PDR, because the blood vessels cannot obtain enough oxygen causing the blood vessels to grow in different regions of the retina images for maintaining the adequate oxygen. Naturally, these blood vessels are prone to leakage, which may lead to vision lost. In this paper, we mainly focus on the detection of red lesions containing microaneurysms and hemorrhages, which are the earliest lesions and more complicated compared to other kinds of lesions detection in the DR.

Numerous approaches have been proposed for red lesion detection. Among them, the earliest paper based on MA detection was proposed by Baudoin et al. [7], which utilized mathematical morphology approach to detect the microaneurysms in fluorescein angiography images of the fundus. After that, two variants of morphological top-hat transformation methods for detecting MAs within fluorescein angiograms were developed by Spencer et al. [8] and Frame et al. [9]. Although the fluorescein angiograms can improve the contrast between the fundus and their background, the usage of intravenous contrast agents is not applicable for everyone, especially the pregnant woman [10]. Therefore, fluorescein angiograms cannot be widely used in public DR screening programs and the adaption of digital color fundus photographs is a better choice for screening purpose.

A number of algorithms based on filtering have been proposed for red lesion detection in digital color fundus photographs. Quellec et al. [11] adopted a template matching method based on subbands of wavelet transformed images for microaneurysm detection, which can solve the uneven illumination or high-frequency noise problems. Besides, Zhang et al. [12] employed Multiscale Gaussian Correlation Coefficients (MSCF) by computing the correlation coefficient between the grayscale distribution of microaneurysms and the Gaussian function to detect MAs. However, MAs and hemorrhages have large variations in their appearance, for example, shape and size. Therefore, how to design a suitable template to match them becomes a vital problem.

Apart from the above-described detection approaches, several mathematical morphology based approaches have been proposed for the detection of red lesions. Júnior and Welfer [13] designed a five-stage detection approach using mathematical morphology for detecting microaneurysms and hemorrhages, obtaining a sensitivity of 87.69% and a specificity of 92.44%. Ravishankar et al. [14] put forward an automatic feature extraction method for multiple lesions detection. In their method, geometrical relationships of different features and lesions can be used along with simple morphological operations. Jaafar et al. [15] suggested a combination of mathematical morphology and rule-based classification approach for the detection of red lesions, which achieved a sensitivity of 86.20% and a specificity of 98.80%. However, the above-mentioned mathematical morphology based approaches heavily depend on the choosing of structuring elements, and it may weaken the performance of algorithms when changing their size and shape. To address this limitation, some pixel classification based methods have been proposed for the detection of red lesions in color fundus photographs [16–20]. Niemeijer et al. [16] incorporated morphological top-hat transform with a *k*NN classifier into a unified framework for automatic detection of red lesions. After that, Sánchez et al. [17] employed the Gaussian mixture model and logistic regression classification for the detection of red lesions. Furthermore, Zhang et al. [18] designed a microaneurysm detection approach, which integrates the dictionary learning (DL) with Sparse Representation Classification (SRC). The microaneurysms can be identified by calculated reconstruction error. Considering the importance of multifeature, Zhou et al. [19] presented a novel approach which combines the multiple features with dictionary learning for microaneurysm detection. The total reconstruction error of multifeature is used for microaneurysm classification. Besides, a microaneurysm detection approach using Sparse PCA and *T*^2^ statistic is developed in [20]. Since their approach just needs to learn one class data, the class imbalance problem can be addressed.

All the above-mentioned detection approaches regard image pixels as the basic unit to distinguish red lesions from nonred lesions. However, image pixels are a consequence of the discrete representation of images but not natural entities. Compared to the pixel-based image representation, superpixel-based image representation is more consistent with human visual cognition and contains less redundancy [21–23]. The reason lies in the fact that superpixel groups pixels into perceptually meaningful atomic regions with similar color and texture and has the ability to adhere to image boundaries. Therefore, it can not only provide a convenient manner to compute image features but also greatly reduce the complexity of subsequent image processing tasks. However, how to detect the true red lesions from a series of superpixel segmentation results is still a problem.

To overcome the above-mentioned issues, in this paper, we propose a novel red lesion detection approach based on superpixel Multichannel Multifeature (MCMF). Two main contributions are as follows: on one hand, a novel candidate extraction scheme based on superpixel segmentation is given, which is more consistent with human visual cognition and contains less redundancy, improving efficiency and accuracy of subsequent image processing tasks. On the other hand, extensive features extracted from multiple-channel images have been proposed and introduced to our feature extraction process, which can improve the performance of red lesion detection.

Our proposed approach consists of the following five phases: first of all, preprocessing is used to make red lesions more visible. Next, candidates can be extracted by applying superpixel segmentation in digital color fundus photographs. And then, these candidates are characterized using not only intensity features in multichannel images (here, “multichannel” means that a series of images can be produced by different operations based on the original image), but also the contextual feature. Besides, Fisher Discriminant Analysis (FDA) [24] is used to perform the classification of candidates. Finally, a postprocessing technique is applied to distinguish red lesions from nonlesions appearing as red such as fovea and blood vessels. Experiments on DiaretDB1 database [25] demonstrate the effectiveness of our proposed method.

The remainder of the paper is organized as follows.  Section 2 describes the proposed MCMF red lesion detection algorithm. The proposed approach is verified in experiments and the corresponding experimental results are reported in Section 3. We end with the conclusion in Section 4.

## 2. The Proposed Method

**Figure 2:**
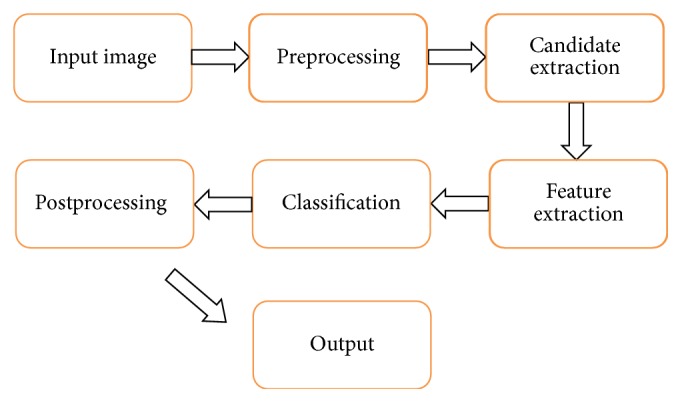
A system description of the proposed approach.

In this section, we mainly introduce the proposed MCMF method for the detection of red lesions.  Figure 2 depicts a flowchart for our proposed approach, which consists of the following five stages: preprocessing, candidate extraction, feature extraction, classification, and postprocessing. Each stage will be described in detail in the following subsections.

### 2.1. Preprocessing

**Figure 3:**
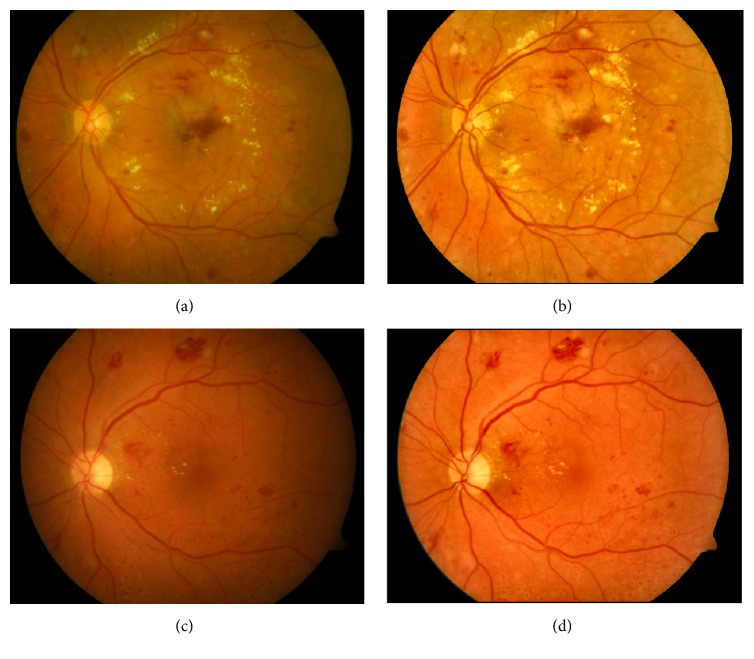
From left to right: original RGB color retinal images (a) and (c); enhancement images (b) and (d).

The large luminosity, poor contrast, and noise always occur in retinal fundus images [11], which affect seriously the diagnostic process of DR and automatic detection of lesions, especially for red lesions. In order to address these problems and make a suitable image for red lesion detection, contrast limited adaptive histogram equalization (CLAHE) [26] method is applied to make the hidden features more visible. Besides, Gaussian smoothing filter with a width of 5 and a standard deviation of 1 is also incorporated for reducing the effect of noise further. Two instances for describing an improvement in color saturation and contrast between lesions and background are shown in Figure 3.

### 2.2. Candidate Extraction

Given a preprocessed image *I*, firstly, SLIC transforms the image *I* into CIELAB color space. Assume that there are *P* pixels in the image and the number of initialized cluster centers is *k*; the grid interval *S* is defined as $S=\sqrt{P/k}$. Secondly, the distance between pixels and the cluster centers should be calculated in each 2*S* × 2*S* region. Here, SLIC combines the color and pixel positions with the cluster centers [*l*, *a*, *b*, *x*, *y*]^*T*^ to compute the distance, where [*l*, *a*, *b*]^*T*^ is three parameters from CIELAB color space and [*x*, *y*]^*T*^ is the position of the pixels. The distance of the pixel *i* to the *k*th cluster center can be defined as in the following.

(1) Dik=dlab2+mS2dxy2dlab=li−lk2+ai−ak2+bi−bk2dxy=xi−xk2+yi−yk2,

where *m* is the weight factor and its range varies from 1 to 40 [21] and (*l*_*i*_, *a*_*i*_, *b*_*i*_) is the color values of the pixel *i* at the position of (*x*_*i*_, *y*_*i*_) of the image *I*.

**Figure 4:**
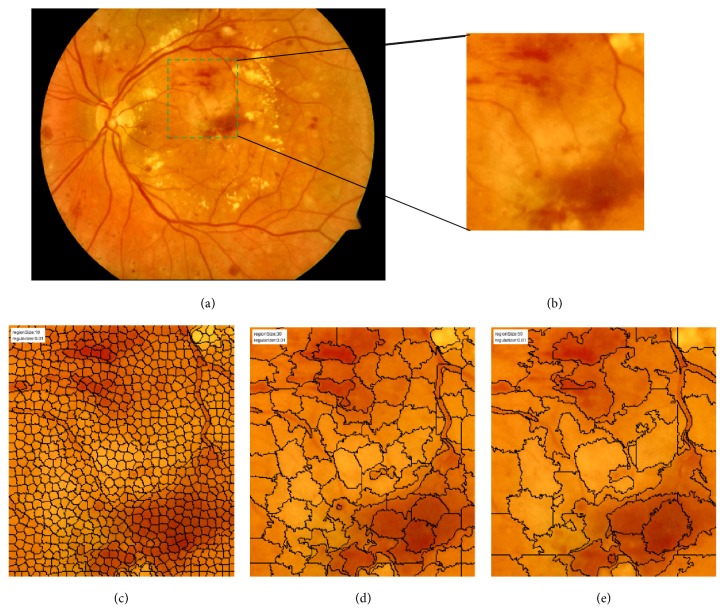
Retinal image segmentation using SLIC. (a) Original retinal fundus image; (b) detail from part of (a); (c)–(e) superpixel segmentation with region sizes 10, 30, and 50 pixels, respectively.

Region size means the size of superpixel segmentation, which plays a vital role in SLIC. For larger region sizes, spatial distances overweigh color proximity, causing the obtained superpixels to not adhere well to image boundaries. For smaller region sizes, the converse is true [21]. Considering the red lesions always vary in size, we will choose a suitable size as our segmentation standard which will be discussed in our experiments.  Figure 4 gives the superpixel segmentation results with different region sizes.

### 2.3. Feature Extraction

As for red lesion detection, some specific properties need to be considered. Firstly, some red lesions are very near the blood vessels, making it hard to distinguish them just in traditional RGB color space. Moreover, the appearance of red lesions is similar to the normal structures of the retinal, such as the blood vessels and the fovea, causing too much false positive (FP). Finally, the shape and size of red lesions especially for large hemorrhages are varied. Based on these facts, we adopt the strategy of extracting different features based on multichannel images for each candidate in this paper. The chosen multichannel images are listed as follows.

**Figure 5:**
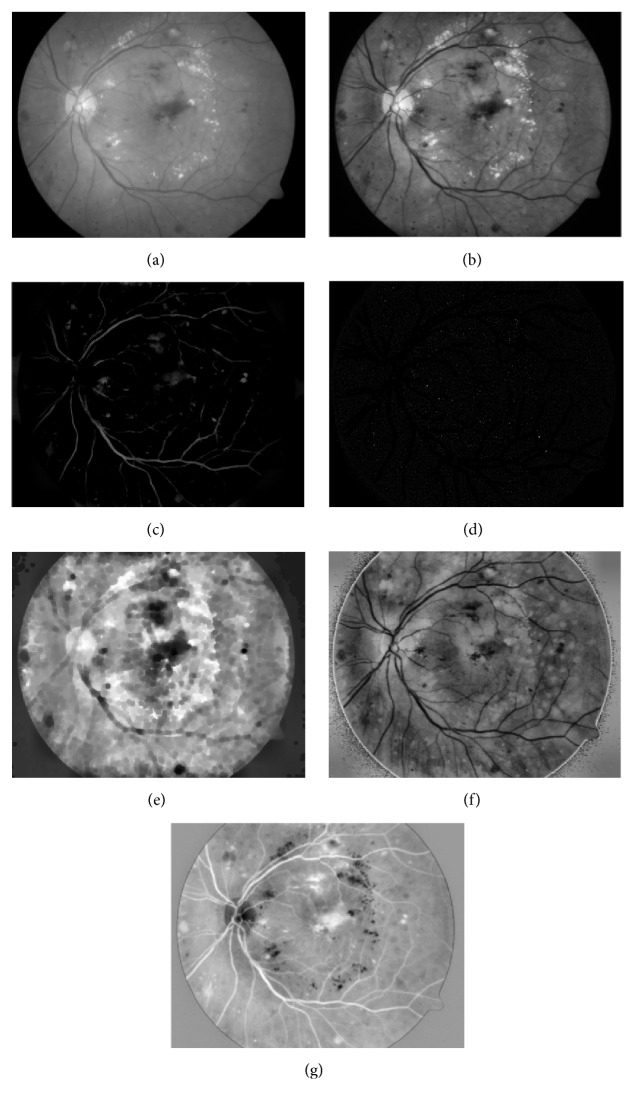
Multichannel images. (a) Original green channel image *I*_*G*_, (b)–(g) enhanced green channel image *I*_green_, enhanced low intensity structure image *I*_dark_enhanced_, *I*_lesions_, close operation image *I*_close_, enhanced hue image *I*_Hue_enhanced_, and *M* component image *I*_*M*_ of CMYK color space, respectively.

*Channel_1-Channel_2.* Green channel image *I*_*G*_ of the original image *I*_original_ and enhanced green channel image *I*_green_ are obtained after preprocessing (see Figures 5(a) and 5(b)).

*Channel_3.* Alternating Sequential Filtering (ASF) containing a set of morphological closing *γ* and opening *ϑ* operations with different sizes of disc-shaped structuring element *K* (10, 20 and 40) is used to estimate the background of preprocessing image in terms of (2). The image of preprocessing is removed from *f*_ASF_ and we will obtain the result image *I*_dark_enhanced_ according to (3). A typical result of this operation is illustrated in Figure 5(c).

(2) fASF=ϑnK⋯γ2KϑKγKIgreen⋯

(3) Idark_enhanced=fASF−Igreen

*Channel_4.* Median filtering with a 25 × 25 pixel kernel to *I*_green_ is used for calculating background image *I*_bg_ and then *I*_bg_ can be removed from *I*_green_ to obtain the shade correlation image *I*_sc_. A series of morphological open operations using 12 line structures of length 9 pixels with different angles ranging from 15 degrees to 165 degrees with the increase of 15 are applied to *I*_sc_ for locating blood vessels. By combining the maximum pixel value at each pixel position in all 12 images, the blood vessels can be obtained. These blood vessels are then subtracted from *I*_sc_ to form *I*_lesions_ containing mainly red lesions with small size. The result of *I*_lesions_ is depicted in Figure 5(d).

*Channel_5.* Morphological close operation [27] is applied to *I*_green_ for eliminating shrinks or thin objects by using a disc-shaped structuring element *B* with radius of 10 pixels; the result image *I*_close_ is illustrated in Figure 5(e).

*Channel_6. I*_Hue_enhanced_ can be obtained by extracting the hue channel image of preprocessing image (see Figure 5(f)).

*Channel_7. I*_*M*_ is *M* component image of the preprocessing image in CMYK color space. The dark structures such as red lesions, blood vessels, and the fovea in *I*_*M*_ are shown as white. On the contrary, the bright structures such as exudates and the optic disc appear as black (see Figure 5(g)).

For each of the above-described multichannel images, four kinds of statistic features including the maximum, minimum, mean, and median are extracted from each candidate. Besides, the total average intensity and standard deviation of the each preprocessing retinal image are also imported.

Since red lesions appear as darker regions with brighter surroundings, based on this characteristic, we also develop a novel and effective feature for distinguishing a candidate from its surroundings and background by mean intensity and the barycenter distance between the neighbor candidates. Here, let av_*G*_*i*_ and *p*_*i*_ denote the mean color in original green channel and barycenter position of the *i*th candidate (*R*_*i*_, *i* = 1,2,…, *N*), respectively. The proposed contextual feature is listed as follows:

(4) Si=∑j∈Niav_Gi/av_Igreen×d1/d2Nid1=av_Gi−av_Gj22if av_Gi≤av_Gj−av_Gi−av_Gj22if av_Gi>av_Gjd2=pi−pj22=∑Im∈RiImPRi−∑Im∈RjImPRj22j∈Ni,

where ‖·‖_2_^2^ denotes a quadratic term of *l*_2_-norm, *N*(*i*) represents the set of neighbors of candidate *i*, and *N*_*i*_ is a total of pixels of candidate *i*. *I*_*m*_^*P*^ is the barycenter position vector, constituted by position vector of pixel *I*_*m*_. *d*_1_ is the mean intensity difference and *d*_2_ is the barycenter positions difference between candidate *i* and its neighbor candidate *j*. Here, the neighbor is defined as the 7 times of the region size empirically.

Basically speaking, our proposed contextual feature needs to calculate the mean gray value of each candidate av_*G*_*i*_ with the aim of making the feature more stable and precise for red lesions. Global mean of image av_*I*_green_ is imported for eliminating the influence caused by the varying retinal pigmentations and different image acquisition processes. If the value of *d*_1_ is positive and large, current candidate is more likely to be a true red lesion, and the converse is false. Besides, the value of *S*_*i*_ is also affected by the barycenter positions distance *d*_2_ between candidate *i* and candidate *j*. From (3), it is clear that a larger *S*_*i*_ indicates that the current candidate more likely belongs to red lesions, and vice versa.

In summary, thirty-one features are computed on each candidate and they can be represented as a vector in a 31-dimension feature set *F* = {*f*_1_, *f*_2_,…, *f*_31_}. Since the different features *f*_*i*_ always varied in values and ranges, we normalize each feature for all the candidates to have zero mean and unit variance by using the following:

(5) $$\begin{array}{c} f_{i}^{\prime }=\frac{f_{i}-\mu_{i}}{\sigma_{i}}, \end{array}$$

where *μ*_*i*_ is the mean of the *i*th feature and *σ*_*i*_ is its standard deviation.

### 2.4. Classification

In this section, Fisher Discriminant Analysis (FDA) [24] is applied to perform the classification of the red lesion candidates. The basic idea of FDA is to seek a transformation matrix which maximizes the between-class scatter and minimizes the within-class scatter simultaneously. Assume a labeled candidate dataset matrix *X* = {*X*_1_, *X*_2_}, where *X*_1_ = {*x*_1_, *x*_2_,…, *x*_*n*_1__} and *X*_2_ = {*x*_1_, *x*_2_,…, *x*_*n*_2__}, the matrix *X*_*k*_  (*k* ∈ {1,2}, 1 = red, 2 = non-red) is from *R*^*d*×*n*_*k*_^,  *d* denotes corresponding feature dimension, and *n*_*k*_ is the total number of samples (*n* = *n*_1_ + *n*_2_) in the *k*th class.

Let *S*^*b*^ and *S*^*w*^ be the between-class scatter matrix and within-class scatter matrix:

(6) Sb=∑k=12nkμk−μμk−μTSw=∑k=12 ∑xi∈Ckxi−μkxi−μkT,

where *μ*_*k*_ = (1/*n*_*k*_)∑_*x*_*i*_∈*C*_*k*__*x*_*i*_ is the mean vector of the *k*th class and *μ* = (*X*_1_ + *X*_2_)/*n*  is the mean vector of the whole candidate dataset.

FDA transformation matrix *W* can be found by maximizing the following optimization problem:

(7) $$\begin{array}{c} W=\frac{w^{T}S^{b}w}{w^{T}S^{w}w}. \end{array}$$

The above optimization problem can be regarded as the generalized eigenvalue problem below [28]:

(8) $$\begin{array}{c} S^{b}\varphi =\lambda S^{w}\varphi , \end{array}$$

where *λ* is the generalized eigenvalue and the vector *φ* is the corresponding eigenvector which is one of columns of the FDA transform matrix *W*.

Since the projected class means *W*^*T*^*μ*_1_ and *W*^*T*^*μ*_2_ are well separated [24], we can choose average of the two projected means as a threshold for classification. In this case, the threshold parameter *c* can be found:

(9) $$\begin{array}{c} c=\frac{W^{T}\left(\mu_{1}+\mu_{2}\right)}{2}. \end{array}$$

Given a new sample *x*, it belongs to class 1, if classification rule *W*^*T*^*x* > *c*, or else it belongs to class 2.

### 2.5. Post-Processing

After the classification stage, some nonlesions appearing as red such as blood vessels and fovea are often present and may be erroneously detected as red lesions. In order to avoid this problem, a postprocessing stage is incorporated into our approach for removing them and improving the robustness of the proposed method.

#### 2.5.1. Multiscale Blood Vessels Detection

Since red lesions and blood vessel have the similar appearance, it is hard to distinguish them effectively. Besides, the red lesions cannot occur on the blood vessels [12]. In order to remove any possible nonlesions caused by blood vessels, a multiscale morphological blood vessel extraction method (MSM) based on [13] is modified. Here, multiscale consists of four different sizes of disc-shaped structure element such as scale = [2 3 4 5] according to [29]. For each scale, we conduct Steps 1to 5 (scale_num is the number of disc structure elements and scale_num equals 4) to extract blood vessels map and the final blood vessels map can be obtained by fusing the blood vessels segmentation maps under all the scales. More details are listed as follows.

**Step 1 .**

Morphological opening *ϑ* and closing *γ* operations with structuring element *K* = scale  (*i*), (*i* represents the *i*th iteration, *i* = 1,2, 3,4, such as scale  (1) = 2 and scale  (4) = 5) are used to estimate the background of preprocessing image *I*_CLAHE_ according to (10) and the result *f*_1_ can be obtained.

(10) $$\begin{array}{c} f_{1}=\gamma^{\left(K\right)}\left(\;\vartheta^{\left(K\right)}\left(\;I_{CLAHE}\;\right)\;\right). \end{array}$$

**Step 2 .**

**Figure 6:**
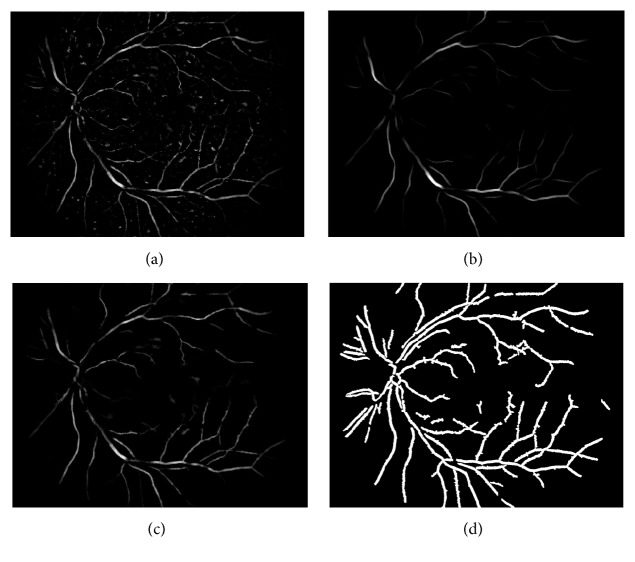
A series of morphological operations are used for blood vessels detection. (a) *f*_2_, reduction of high intensity structures; (b) *f*_3_, sum of morphological openings; (c) *f*_4_, morphological reconstruction by dilation; (d) *f*_5_^scale^, regional minimum and close operation (e.g., we take scale  (4) = 5).

The high intensity structures can be eliminated by subtracting the CLAHE result image from *f*_1_; see Figure 6(a).

(11) $$\begin{array}{c} f_{2}=f_{1}-I_{CLAHE}. \end{array}$$

**Step 3 .**

Morphological opening with varying structuring elements is applied to *f*_2_ for locating blood vessels. Here, we use the linear structuring elements *ψ* with 12 different angles ranging from 15 degrees to 165 degrees with the increase of 15. By accumulating the pixel values at each pixel position in all 12 images, we can obtain the image *f*_3_ according to the following (see Figure 6(b)).

(12) $$\begin{array}{c} f_{3}=\psi^{1}f_{2}+\psi^{2}f_{2}+\cdots ,\psi^{12}f_{2}. \end{array}$$

**Step 4 .**

According to (13), a morphological dilation reconstruction is used to detect the blood vessels furthermore, which is denoted by *R* (see Figure 6(c)).

(13) $$\begin{array}{c} f_{4}=R\left(\;f_{3}\;\right). \end{array}$$

**Step 5 .**

A binary vessel structure map *f*_5_^scale^ can be obtained by combining morphological operator of regional minimum RM with close operation *R*_close_ according to the following (see Figure 6(d)).

(14) $$\begin{array}{c} f_{5}^{scale}=R_{close}\left(\;-RM\left(\;f_{4}\;\right)\;\right) \end{array}$$

Repeat Steps 1to 5 until the value of scale reaches the maximum iteration number (scale_num).

**Figure 7:**
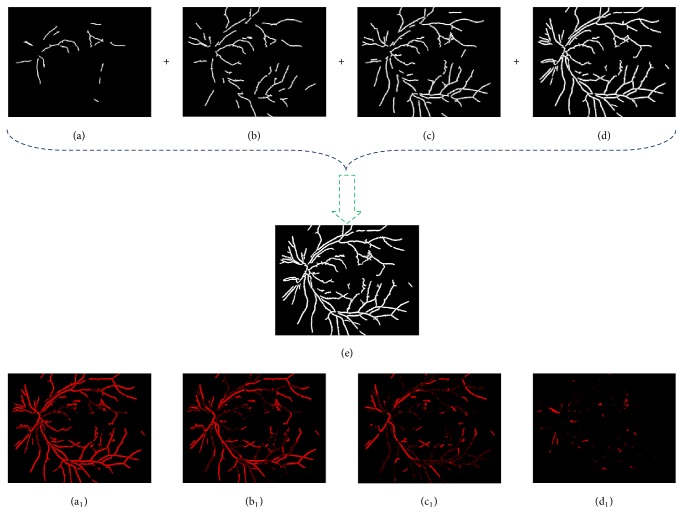
Blood vessels extraction results. (a)–(d) show the different detection results with varying sizes of disc-shaped structuring elements 2, 3, 4, and 5, respectively; (e) the combination result of (a)–(d); (a_1_)–(d_1_) are the blood vessels map differences between (e) and (a)–(d), respectively.

At last, we will obtain the final blood vessels map *f*_5_ by combining each of the blood vessels segmentation maps with the logical OR operation under all the scales (see Figures 7(a)–7(d)) (“|” represents logical OR operation and the entire vessel network is shown in Figure 7(e)).

(15) $$\begin{array}{c} f_{5}=f_{5}^{1}\left|\;f_{5}^{2}\;\right.\cdots \left|\;f_{5}^{scale\_num}\;\right.. \end{array}$$

The main difference between our proposed MSM blood vessels extraction method and the single scale blood vessels detection method [13] lies in whether to take the multiscale into consideration or not. Figures 7(a)–7(d) show the blood vessels maps produced by [13] and Figures 7(a_1_)–7(d_1_) are the difference images by subtracting the images in Figures 7(a)–7(d) from MSM detection result shown in Figure 7(e). Comparing with these results, we can learn that our proposed approach achieves a better robust and complete blood vessels segmentation result than the single scale blood vessels detection [13], which can do well in reducing the FP in red lesion detection.

#### 2.5.2. Elimination of the Fovea

**Figure 8:**
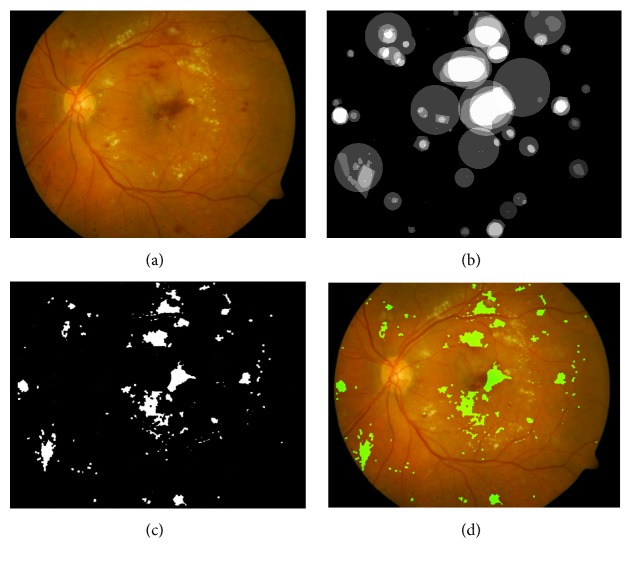
The final result of our proposed MCMF. (a) Original retinal image; (b) the corresponding ground truth; (c) final red lesions map; (d) overlaying red lesions map on original retinal image.

The fovea appears as dark region located in the center of the macula region of the retina. Since the fovea region has a relative low intensity profile and its appearance is commonly much similar with the background causing it to be hard to distinguish from true red lesions. In order to improve the accuracy of the proposed method, the removal of fovea is indispensable. A method [16] by considering the spatial relationship between the diameter of the optic disc and the region of the fovea is adopted to remove the fovea. The final red lesion map result is shown in Figure 8.

**Figure 9:**
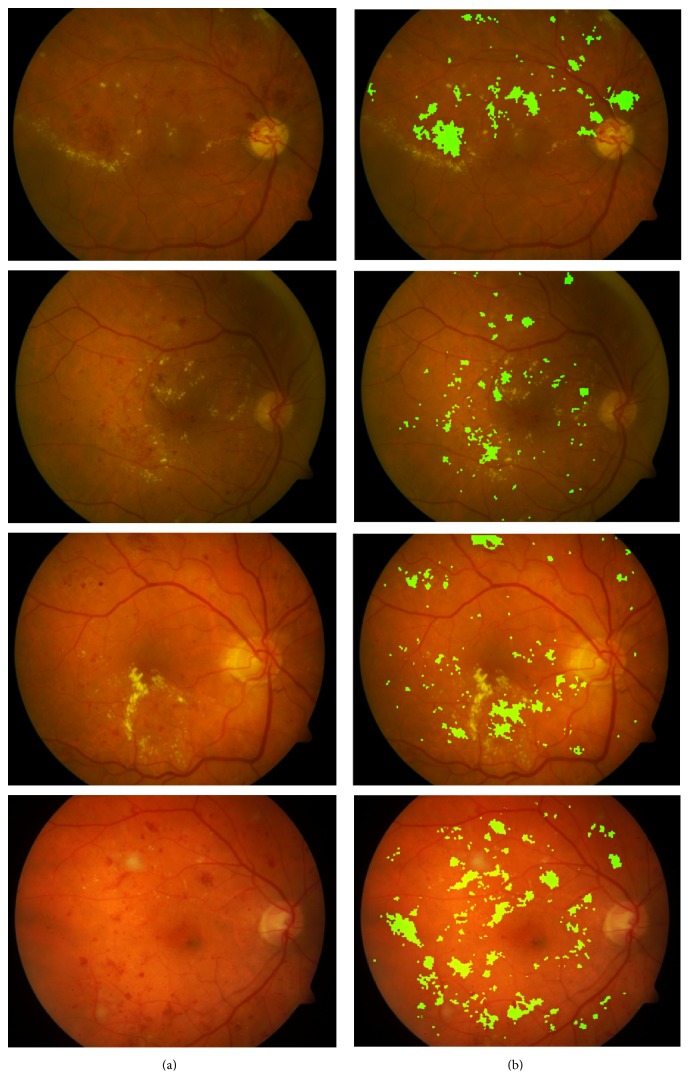
Results of our proposed red lesion detection approach on abnormal retinal images. (a) Original retinal images; (b) the corresponding red lesions maps.

Figure 9 depicts more examples for our proposed MCMF approach for red lesions detection in the retinal images containing red lesions.  Figure 9(a) shows original color retinal images; Figure 9(b) illustrates their detected red lesion maps. From these results, we can learn that our proposed MCMF red lesion detection approach can detect most of red lesions in the retinal images regardless of their varying sizes and shapes.

## 3. Experimental Results and Analysis

### 3.1. Database Description

In this section, we conduct extensive experiments to validate and evaluate the effectiveness of our proposed red lesion detection method on public DiaretDB1 retinal image database [25].

The DiaretDB1 database (Standard Diabetic Retinopathy Database Calibration level 1, version 1) [25] is a public database available on the web. The database contains a total of 89 RGB color fundus images with the fixed 1500 × 1152 resolution and 50° field of view. Among the 89 fundus images, 5 images are healthy and the remaining 84 retinal images are abnormal, which are annotated by four different clinical experts. In our experiment, the training set consists of 40 retinal images selected randomly. The testing set is composed of the remaining 49 images.

### 3.2. Assessment of Classification Performance

We take two evaluation criterions to verify the effectiveness of our proposed red lesion detection method, such as sensitivity and specificity. These measures are calculated based on the given red lesion ground truth, which can be seen from the following:

(16) sensitivity=TPTP+FNspecificity=TNTN+FP,

where true positive (TP) is the number of red lesions that are correctly identified, false negative (FN) is the number of lesions incorrectly found as nonred lesions, false positive (FP) is the number of lesions incorrectly found as red lesions, and true negative (TN) is the number of nonred lesions that are correctly identified. These criteria are also used to evaluate the performance of different methods for the detection of red lesions.

In our experiments, we employ the Receiver Operating Characteristics (ROC) curve to evaluate the effectiveness of the proposed red lesion detection method. An ROC curve is the plot of sensitivity on the vertical axis and (1 − specificity) on the horizontal axis. In ROC curve, the upper left corner represents perfect classification. Besides, the area under the ROC curve is denoted as the AUC value for measuring and describing the algorithm performance. A larger AUC value indicates a better classifier performance.

### 3.3. Results

In this section, we will carry out three experiments based on DiaretDB1 dataset to verify the effectiveness of our proposed method. In our experiments, we employ two different kinds of criteria to evaluate the method performance, including image-based [25] and pixel-based [30] criteria. Image-based criterion means to classify an image either as “normal” or “abnormal” (i.e., detecting the absence or presence of red lesions anywhere in the image). That is to say, a retinal image is considered as pathological if it presents one or more red lesions; otherwise it is normal [22]. However, in pixel-based criterion, we adopt the connected component level validation [28] by counting the number of pixels detected correctly. According to [28], connected components are considered as true positive (TP) if they totally or partially overlap with the ground truth (i.e. a red lesion is considered as TP if the detected connected component overlapped at least 75% of the area manually annotated by the expert but less than 100%, and all other conditions are considered to be false detection). The TN, FP, and FN are calculated in the same manner. Based on these observations, algorithm validated based on the image-based criterion typically achieves a better performance than the pixel-based criterion since it is not necessary to detect all the red lesions in the images. For more details refer to [30]. Taking the clinical point of view and screening applications into consideration, it is more interesting to evaluate the experiment results at image-based criterion [31].

There is a parameter in our proposed method, region size (the size of superpixel), which impacts the performance of our proposed method. How to choose a suitable size becomes a critical problem in our experiment. Besides, for the same test sample, different classifiers may obtain the different classification results. So the choosing of classifier is equally important.

**Figure 10:**
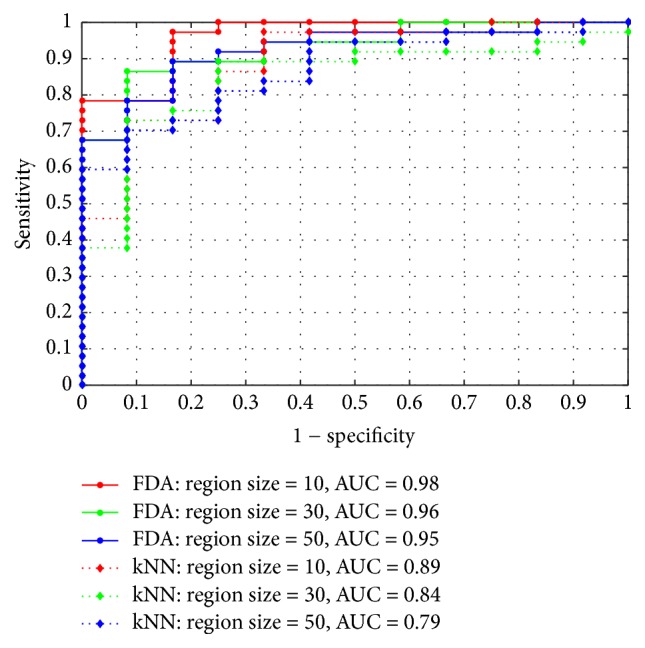
Sensitivity versus 1 − specificity curves with varied region sizes by applying two different classifiers.

In our first experiment, two supervised classifiers, FDA and *k*NN (*k* nearest neighborhood) [32], as the underlining classifiers are selected and the optimal one will be used for our following experiments. Here, we set *c* as 0.01 for FDA classification according to (9). Three different region sizes (10, 30, and 50) as our segmentation standards are used to select the optimal classifier based on image-based criterion. Firstly, training samples with three different region sizes are used to train corresponding classifiers containing FDA and *k*NN, respectively; and then we adopt the trained classifiers to classify test samples with various region sizes and obtain corresponding classification scores. The experiment results are shown in Figure 10.

According to Figure 10, we can learn that FDA classifier can achieve the better results than *k*NN classifier in all the region sizes. The reasons are listed as follows: since the *k*NN classifier works by comparing the Euclidean distance of a test sample with *k* labeled training samples, which are also known as *k* nearest neighbors on the whole feature space, due to the original feature space containing some irrelevant or redundant features, which causes misclassification for *k*NN. Different from *k*NN, FDA is a well-known linear technique for dimensionality reduction and feature extraction, which can avoid the above problem greatly. FDA makes full use of the label information to find the optimal projection vectors by maximizing between-class scatter matrix and minimizing within-class scatter matrix simultaneously and achieves a better classification performance. Therefore, the FDA will be chosen as our optimal classifier for the following experiments.

Besides, judging from Figure 10, we can see that when the region size is set to 10, our proposed algorithm achieves the best performance both in FDA and in *k*NN. Yet, we can also notice that, for each kind of classifiers, the different choosing of region sizes is not sensitivity for our classification result, which can achieve quite similar ROC curves.

**Figure 11:**
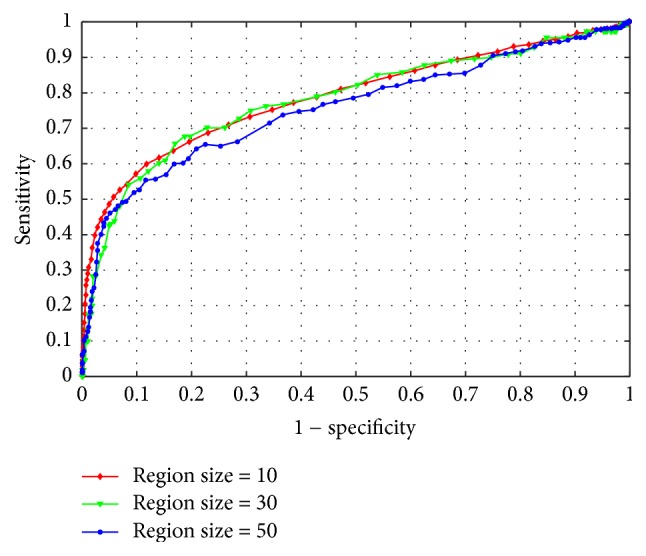
ROC curves of pixel-based criterion on testing set with varying region sizes.

In our second experiment, we will find the optimal region size based on pixel-based criterion [30]. The ROC curves are shown in Figure 11.

Judging from the above experiment results, it is easy to find that the performances of all the region sizes in the pixel-based criterion are relatively lower than those in the image-based criterion. The reason lies in the fact that the range of ground truth provided in database is not very precise and always larger than the true size of red lesions. So when each obtained red lesion map compares with its ground truth, the imprecise ground truth will affect the method's performance. What is more, choosing the smaller size of superpixel, the segmentation results are more accurate than the bigger ones and when the region size is set to 10, our proposed algorithm achieves the best performance (i.e., AUC = 0.74) as shown in Table 1. However, smaller region size leads to higher computing complexity. When the region size = 10, the average execute time for each image approximately takes 116.23 s and the average computing time reaches about 10.37 s for the region size = 50 listed in Table 1 (the average running time of per image is evaluated using Matlab R2015a on a PC with Intel Core i5 running Windows 7 at the clock of 3.30 GHZ with 16 G RAM).

In addition, considering the clinical point of view and for screening applications [31], choosing the bigger region size has completely attended their goals. Taking the above reasons into consideration, we set the region size (superpixel segmentation size) as 50 in our next experiment.

In our last experiment, we employ image-based evaluation method proposed by Kauppi et al. [25] to verify the effectiveness of the proposed method by comparing it with other state-of-the-art methods. According to [25], each image needs to provide a score and a high score means a high probability that a lesion presents in corresponding image. With the provided scores, the sensitivity and specificity measures can be calculated. By image-based comparison, the proposed approach achieves a sensitivity of 83.30% and a specificity of 97.30%.  Table 2 lists several comparison results obtained by the existing red lesion detection methods on DiaretDB1 database.

From the comparison results of various algorithms illustrated in Table 2, we can know that our proposed method is more reliable than the other methods and achieves satisfactory result. But there are still some points that need to be mentioned as follows. Firstly, from Figures 8 and 9, it can be found that the residues of blood vessels close to red lesions cannot be removed from the final red lesion maps causing false positives. Secondly, when there are small size red lesions contained in the retinal images, they may not be completely detected.

## 4. Conclusion

To summarize, we put forward a novel red lesion detection based on superpixel Multichannel Multifeature (MCMF) classification in color retinal images, which is able to detect the red lesions efficiently regardless of their variability in appearance and size. Firstly, the whole image is segmented into a series of candidates using superpixel segmentation. And then, multiple features from the multichannel images as well as the contextual feature are proposed for describing each candidate. Next, FDA is introduced to classify the red lesions among the candidates. Finally, a postprocessing technique is applied to distinguish red lesions from blood vessels and fovea. Experiment results on DiaretDB1 database demonstrate that our proposed method is effective for red lesion detection.

Since our proposed approach extracts a number of features for each superpixel, complex relationships among the extracted features exist and other classifier (e.g., neural network or Extreme Learning Machine) may lead better classification result which could be validated and researched in future works. In addition, applying our proposed framework to other lesions detection is also another interesting topic for future study.

## Figures and Tables

**Table 1:** Average execute time of per image and AUC with varying region sizes at pixel-based criterion.

	AUC	Average time in seconds
Region size = 10	0.74	116.23 s
Region size = 30	0.73	38.05 s
Region size = 50	0.70	10.37 s

**Table 2:** Performance results on DiaretDB1 dataset.

Authors	Sensitivity	Specificity
Proposed method	83.30%	97.30%
Sánchez et al. [17]	87.69%	92.44%
Ravishankar et al. [14]	95.10%	90.50%
Jaafar et al. [15]	98.80%	86.20%
Roychowdhury et al. [33]	75.50%	93.73%
